# Carbon‐ion radiotherapy combined with chemotherapy for head and neck mucosal melanoma: Prospective observational study

**DOI:** 10.1002/cam4.2614

**Published:** 2019-10-16

**Authors:** Yukihiro Takayasu, Nobuteru Kubo, Masato Shino, Osamu Nikkuni, Shota Ida, Atsushi Musha, Katsumasa Takahashi, Junko Hirato, Katsuyuki Shirai, Jun‐ichi Saitoh, Satoshi Yokoo, Kazuaki Chikamatsu, Tatsuya Ohno, Takashi Nakano, for the Working Group on Head and Neck Tumors

**Affiliations:** ^1^ Department of Otolaryngology‐Head and Neck Surgery Gunma University Graduate School of Medicine Maebashi Gunma Japan; ^2^ Gunma University Heavy Ion Medical Center Maebashi Japan; ^3^ Department of Otolaryngology Takasaki General Medical Center National Hospital Organization Takasaki Japan; ^4^ Department of Pathology Gunma University Hospital Maebashi Japan; ^5^ Department of Radiology Jichi Medical University Saitama Center Saitama Japan; ^6^ Department of Radiology Graduate School of Medicine and Pharmaceutical Sciences University of Toyama Toyama Japan; ^7^ Department of Oral and Maxillofacial Surgery, Plastic Surgery Gunma University Graduate School of Medicine Maebashi Japan

**Keywords:** chemotherapy, head and neck neoplasm, melanoma, radiotherapy, survival

## Abstract

This study aimed to evaluate the efficacy of carbon‐ion radiotherapy in combination with chemotherapy using dacarbazine, nimustine, and vincristine (DAV therapy) in mucosal melanoma. Twenty‐one patients with clinically localized mucosal melanoma of the head and neck were enrolled. The primary endpoint was 3‐year overall survival (OS). Secondary endpoints included local control, progression‐free survival (PFS), and adverse event occurrence. Carbon‐ion radiotherapy with a dose of 57.6‐64.0 Gy (relative biological effectiveness) in 16 fractions was delivered concurrently with DAV therapy, and 2 cycles of adjuvant DAV therapy were administered every 6 weeks. The median follow‐up periods were 15.5 months for all patients, and 31.2 months for 12 surviving patients. All patients had locally advanced T4a or T4b disease in the rhino‐sinus area. In 16 patients (76.2%), 3 cycles of planned DAV therapy were completed. The 3‐year OS and PFS rates were 49.2% and 37.0% respectively. The 3‐year local control rate was 92.3%. Eleven patients (52%) developed distant metastasis, which was the most frequent pattern of the first failure. Commonly presenting acute grade 2‐3 toxicities associated with radiotherapy and chemotherapy were mucositis (11 patients [53%]) and leukopenia (9 patients [43%]), which improved with conservative therapy. None of the patients developed grade 3 or greater late toxicities. Carbon‐ion radiotherapy in combination with DAV therapy led to excellent local control for advanced mucosal melanoma within acceptable toxicities. The efficacy of additional DAV therapy in improving survival was weaker than expected as distant metastases still occurred frequently. Trial registration no. UMIN000007939.

## Graphical abstract

It is the first prospective trial to evaluate the clinical outcomes of carbon‐ion radiotherapy combined with chemotherapy.

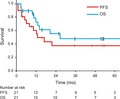

## 1 INTRODUCTION

The incidence of primary mucosal melanoma is very low, at 1‐2 people per million, and about half of all such cases present with the disease in the head and neck region.^1^ The ratio of mucosal melanoma to all head and neck cancers is 1%~ as per the 2002 and 2015 reports of the Head and Neck Cancer Registry of Japan.^2^, ^3^

Mucosal melanoma is an aggressive malignancy with a very poor prognosis, with a 5‐year survival rate of 30% or lower, and low rates of local control and frequently occurring distant metastasis.^1^, ^4^, ^5^ The standard treatment for mucosal melanoma of the head and neck comprises radical surgery and adjuvant radiotherapy.^6^, ^7^ However, the achievement of local control using radical surgery and conventional radiotherapy remains problematic, especially in advanced‐stage disease, as the surgical margins and dose escalations (for radiotherapy) are limited by adjacent critical structures and organs.^8^ Consequentially, even in patients with operable tumors, the 5‐year overall survival (OS) rate is 25%‐46%.^9^

Carbon‐ion radiotherapy has the additional advantage of showing stronger efficacy against relatively radioresistant tumors on the basis of their radiobiological characteristics and highly concentrated dose distributions^10^; thus, it is now a standard curative option for unresectable mucosal melanoma of the head and neck in Japan. Previous studies on carbon‐ion radiotherapy for mucosal melanoma of the head and neck have shown local control rates greater than 80% and OS rates lower than 50% with frequently presenting distant metastasis.^11^, ^12^ These results suggest that the management of distant metastasis is essential in improving survival in such cases.

In Japan, combination chemotherapy including dacarbazine (DTIC), nimustine (ACNU), and vincristine (VCR) (DAV therapy) is frequently adopted for cutaneous melanoma based on domestic clinical trials.^13^, ^14^ Combining such a systemic chemotherapy regimen with carbon‐ion radiotherapy for mucosal melanoma is expected to contribute to improved patient prognoses. Therefore, we performed a prospective trial to establish the efficacy and safety of carbon‐ion radiotherapy with concurrent and adjuvant DAV therapy for mucosal melanoma of the head and neck. To the best of our knowledge, this is the first prospective trial to evaluate the clinical outcomes of carbon‐ion radiotherapy combined with chemotherapy in such settings.

## 2 MATERIALS AND METHODS

### 2.1 Patient and tumor characteristics

All patients with mucosal melanoma of the head and neck were prospectively treated following a protocol for carbon‐ion radiotherapy in combination with systemic chemotherapy, as approved by our Institutional Review Board. The trial was conducted in accordance with the tenets of the Declaration of Helsinki, and is registered with the University Hospital Medical Information Network Clinical Trials Registry (https://www.umin.ac.jp/ctr/index-j.htm), identification number UMIN000007939. Inclusion criteria according to the protocol are: (a) histologically confirmed head and neck mucosal melanoma; (b) clinical T1‐4N0M0 (Union for International Cancer Control staging criteria, 7th edition); (c) measurable tumor; (d) age between 16 and 80 years; (e) reserved normal cardiac, pulmonary, hepatic, and bone marrow function: serum creatinine level ≤1.5 mg/dL, blood urea nitrogen (BUN) level ≤25 mg/dL, creatinine clearance ≥60 mL/minute, total bilirubin level ≤1.5 mg/dL, aspartate transaminase (AST) and alanine transaminase (ALT) level ≤1.5 × institutional upper limit of normal (ULN), white blood cell count ≥4000/mm^3^, platelet count ≥100 000/mm^3^, hemoglobin level ≥10 g/dL and PaO_2_ ≥70 mmHg, and (f) performance status of 0‐2. Exclusion criteria are the following: (a) prior history of irradiation in the head and neck; (b) prior history of chemotherapy within 4 weeks before carbon‐ion radiotherapy administration; (c) severe infection; (d) severe clinical complication; and (e) active multiple primary cancer. All biopsy materials were re‐evaluated by one central pathologist (JH) at Gunma University Hospital (Maebashi, Japan). Evaluations included physical examinations, laryngoscopy, computed tomography (CT), MRI, and 18‐fluorodeoxyglucose PET performed within 1 month before treatment. The primary endpoint was 3‐year OS. Secondary endpoints included local control rate, progression‐free survival (PFS), and adverse event occurrence.

### 2.2 Carbon‐ion radiotherapy

Written informed consent was obtained from all patients before the study. The patients were immobilized in the supine or prone position using thermoplastic shells (Shellfitter; Kuraray) in customized cradles (Moldcare; Alcare). A mouthpiece was used to support the position of mandible. CT images were acquired at 2 mm slice thickness and used for treatment planning. MRI was also performed to assist in target delineation. The XiO‐N system (Elekta) was used for treatment planning. The radiation dose for carbon‐ion radiotherapy was described using the unit of Gy (relative biological effectiveness [RBE]), which was defined as the physical doses multiplied by the RBE of the carbon ions. Our facility had vertical and horizontal irradiation ports with passive beams. Since rotating‐gantry was not available, the patient's seating was rotated if necessary. Delineation of the gross tumor volume (GTV) was determined with reference to contrast‐enhanced MRI. Two clinical target volumes CTV1 and CTV2 were delineated. CTV1 encompassed whole anatomic sites of the tumor origin (eg nasal cavity or maxillary sinus). CTV2 was defined as the GTV plus a 3 mm margin in all directions. Two planning target volumes PTV1 and PTV2 were also created, with these having 2 mm margins around CTV1 and CTV2 respectively. The margins of CTV and PTV were modified if the tumors were adjacent to organs at risks (OARs). The dose was administered to the isocenter of the PTVs. The PTVs were covered by the 95% of the prescribed dose. A total of 64.0 Gy (RBE) was administered in 16 fractions with 4 fractions per week in all cases except one, in which the tumor was close to the skin, due to which 57.6 Gy (RBE) was delivered in 16 fractions. PTV1 was received a total dose of 36 Gy (RBE) and PTV2 received the remaining dose. The dose constraints of the OARs are as following: maximum dose to spinal cord <30 Gy (RBE),^15^ maximum dose to the optic nerve <57 Gy (RBE),^16^ and 60 Gy (RBE) <20 cm^2^ to the skin.^17^

### 2.3 Chemotherapy

**Figure 1:**
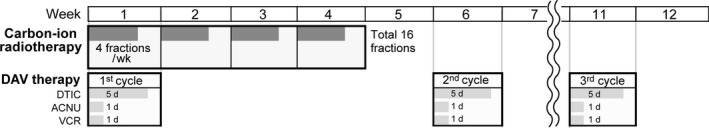
Schematic diagram of treatment schedule of carbon‐ion radiotherapy with concurrent and adjuvant DAV chemotherapy. DAV therapy was administered every 6 wks over 3 cycles. DAV, dacarbazine, nimustine, vincristine

As shown in Figure 1, at the initial week during which carbon‐ion radiation was initiated, all patients received 1 cycle of systematic chemotherapy, combined with DTIC, ACNU, and VCR (DAV therapy). DTIC (120 mg/m^2^/day) was infused intravenously from the first day to the fifth day. ACNU (70 mg/m^2^) and VCR (0.7 mg/m^2^) were intravenously administered on the first day. As adjuvant chemotherapy, DAV therapy was performed every 6 weeks in a total of 3 cycles, if the patient had no serious complications. DAV therapy could be skipped for 1 week in the case of the development of hematotoxicity, nephrotoxicity, or hepatotoxicity: white blood cell count <3000/mm^3^, platelet count <75 000/mm^3^, hemoglobin level <8.0 g/dL, serum creatinine level ≥ 2.0 mg/dL, BUN ≥ 30 mg/dL, creatinine clearance <55 mL/min, total bilirubin level ≥2.0 mg/dL, and AST and ALT level >1.5 × institutional ULN. DAV therapy could be permanently stopped if treatment was skipped for 3 consecutive weeks. All patients were monitored by clinical examinations and blood tests at least once every month over the 6‐month observation period.

### 2.4 Evaluation

Tumor staging was performed on initial diagnostic scans according to the TNM classification 7th edition. Patients had a medical examination every month for the first 6 months and every 3 months thereafter. MRI and CT were performed alternately every 3 months, and 18‐fluorodeoxyglucose PET was conducted every year. Response assessment was performed through follow‐up MRI or CT according to Response Evaluation Criteria in Solid Tumors version 1.1 criteria.

Local control was defined as the absence of further tumor growth after radiation or absence of further tumor growth after the achievement of the best response in the treated lesion(s). Progression‐free survival (PFS) was defined as the absence of local or distant failure or death from any cause.

Toxicities were graded using the Common Terminology Criteria for Adverse Events version 4.0. Weekly follow‐ups were continued until the acute toxicities were easily manageable, and late toxicities were scored every 3 months after irradiation. Acute and late toxicities were defined as events that developed within 90 days and later than 90 days after carbon‐ion radiotherapy initiation respectively.

When the tumor was recurrent during or after the protocol treatment, patients were able to receive any salvage treatments according to the investigator's decision.

### 2.5 Statistical analysis

Time‐to‐event data were calculated from the start of carbon‐ion radiotherapy to the last follow‐up date or death. Local control, PFS, and OS were estimated using the Kaplan‐Meier method, and the potential prognostic factors (age, gender, T‐stage, DAV cycle) for PFS and OS were evaluated using log‐rank tests. Statistical analyses were performed using BellCurve for Excel (Social Survey Research Information Co., Ltd) and SPSS, version 21 (IBM).

## 3 RESULTS

Between July 2012 and January 2019, 21 patients with mucosal melanoma of the head and neck prospectively underwent carbon‐ion radiotherapy combined with DAV therapy at Gunma University Heavy Ion Medical Center. Their median age was 66 years (range: 32‐80 years). This trial was closed after 21 patients were enrolled over a six‐and‐a‐half‐year period due to poor accrual, owing to the low incidence of mucosal melanoma and competition associated with novel treatments for melanoma using immune checkpoint inhibitors. The patients’ characteristics and therapeutic outcomes are shown in Table 1. All patients showed very advanced disease (T4a: 19 patients, 90.5%; T4b: 2 patients, 9.5%), and the predominantly observed tumor sites were the nasal cavity (16 patients, 76.2%) and maxillary sinus/gingiva (5 patients, 23.8%), all of which were included in the rhino‐sinus area. All patients were able to successfully complete carbon‐ion radiotherapy. The prescribed dose of carbon‐ion radiotherapy was 64.0 Gy (RBE) in 20 cases, and 57.6 Gy (RBE) in one case in which the tumor margin was close to the skin. Three planned cycles of DAV therapy were completed in most cases (16 patients: 76.2%). In the other 5 patients (23.8%), only one or two cycles of DAV therapy were performed. The reason was the early development of distant metastasis in 2 patients, local recurrence in 1 patient, and time to recovery from acute grade 3 leukopenia longer than 3 weeks in 2 patients.

**Table 1:** Patient characteristics and therapeutic outcomes

Patient no.	Tumor site	T Stage	DAV cylces	Local recurrence	Distant metastasis	Additional treatments after recurrence	Survival
1	Nasal cavity	4a	3	No	None		Alive
2	Nasal cavity	4a	3	No	Lung, brain, adrenal gland	γ‐Knife (for brain meta)	Dead
3	Maxillary sinus	4a	2	No	Lung	None	Dead
4	Nasal cavity	4a	3	No	Lung, skin, bone	Chemotherapy^a^, ^a^	Dead
5	Maxillary sinus	4a	1	No	Brain	None	Dead
6	Nasal cavity	4a	3	No	None		Alive
7	Maxillary gingiva	4a	3	No	None		Alive
8	Nasal cavity	4a	3	No	Liver	None	Dead
9	Nasal cavity	4a	3	No	Bone	Nivolumab	Dead
10	Nasal cavity	4a	3	No	None		Dead (of other diseases)
11	Nasal cavity	4a	3	No	None		Alive
12	Nasal cavity	4b	3	No	Bone	None	Dead
13	Nasal cavity	4a	2	No	Bone	PTX + CBCDA →Nivolumab	Dead
14	Nasal cavity	4a	3	No	Liver	Nivolumab	Alive
15	Nasal cavity	4a	1	Yes	None	Salvage surgery	Alive
16	Nasal cavity	4a	3	No	None		Alive
17	Maxillary sinus	4b	3	No	Brain, bone, pancreas	Nivolumab → Ipilimumab	Dead
18	Maxillary sinus	4a	1	No	None		Alive
19	Nasal cavity	4a	3	No	None		Alive
20	Nasal cavity	4a	3	No	None		Alive
21	Nasal cavity	4a	3	No	Bone, skin	Nivolumab	Alive

Abbreviation: CBCDA, carboplatin; DAV, dacarbazine, nimustine and vincristine; PTX, paclitaxel.

a Regimen unspecified.

### 3.1 Toxicity

The major acute and late adverse events that occurred in all 21 patients are listed in Table 2. There were no grade 4 toxicities. The most commonly occurring acute toxicities with regards to carbon‐ion radiotherapy were mucositis, dermatitis and conjunctivitis. Acute radiation mucositis was commonly observed in 53% of the patients (grade 2, 43%; grade 3, 10%). Grade 2 dermatitis also frequently presented (38%); however, no grade 3 dermatitis was evident. In terms of chemotherapeutic toxicity, thrombocytopenia and leukopenia (≤grade 3) were observed in 6 patients (29%) and 9 patients (43%) respectively. Grade 3 leukemia was noted in 5 patients (24%), accounting for the most commonly occurring grade 3 toxicity. The degree of these acute adverse events improved with conservative therapy. Regarding late toxicities such as chronic sinusitis, nasal congestion and otitis media occurred in some patients after several months following carbon‐ion therapy, but no cases with fatal toxicities or severe late toxicities (≥ grade 3) were observed.

**Table 2:** Acute and late adverse events (grade ≥ 2) for all patients (n = 21)

	Grade 2 (%)	Grade 3 (%)	Grade 4 (%)
Acute adverse event			
Mucositis	9 (43)	2 (10)	0 (0)
Dermatitis	8 (38)	0 (0)	0 (0)
Conjunctivitis	4 (19)	0 (0)	0 (0)
Thrombocytopenia	5 (24)	1 (5)	0 (0)
Leukopenia	4 (19)	5 (24)	0 (0)
Anemia	3 (14)	0 (0)	0 (0)
Dysgeusia	1 (5)	0 (0)	0 (0)
Late adverse event			
Mucositis	4 (19)	0 (0)	0 (0)
Dermatitis	0 (0)	0 (0)	0 (0)
Nasal congestion	3 (14)	0 (0)	0 (0)
Nasolacrimal duct obstruction	0 (0)	0 (0)	0 (0)
Sinusitis	3 (14)	0 (0)	0 (0)
Otitis media	3 (14)	0 (0)	0 (0)
Maxilla osteonecrosis	3 (14)	0 (0)	0 (0)
Oronasal/Oroantral fistula	1 (5)	0 (0)	0 (0)

### 3.2 Efficacy

**Figure 2:**
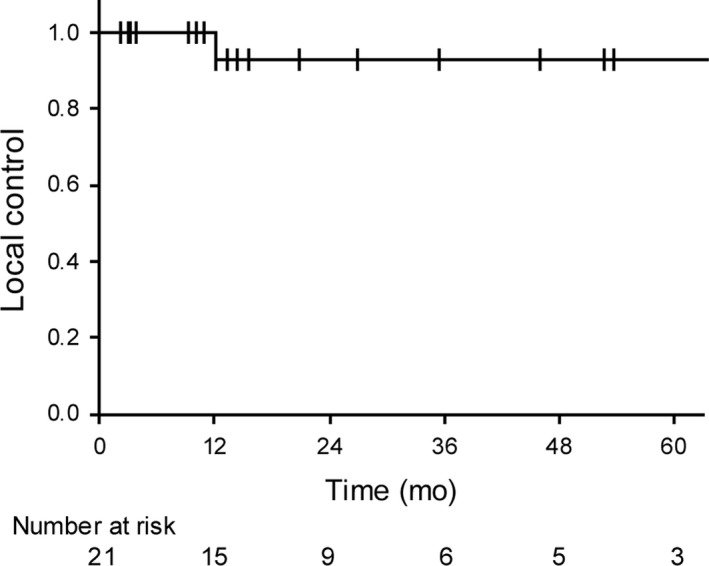
Kaplan‐Meier plots of local control. The 3‐year local control rate for all patients (n = 21) was 92.3%

The median follow‐up periods were 15.5 months (range: 3.0‐76.7 months) for all 21 patients and 31.2 months (range: 3.8‐76.7 months) for the surviving 12 patients. Only 1 patient (5%) developed local recurrence after 12 months from the initiation of carbon‐ion radiotherapy; the patient underwent salvage surgery and is still alive. The 3‐year local control rate for all patients was 92.3% (95% confidence interval [CI]: 79.4%‐100%, Figure 2).

**Figure 3:**
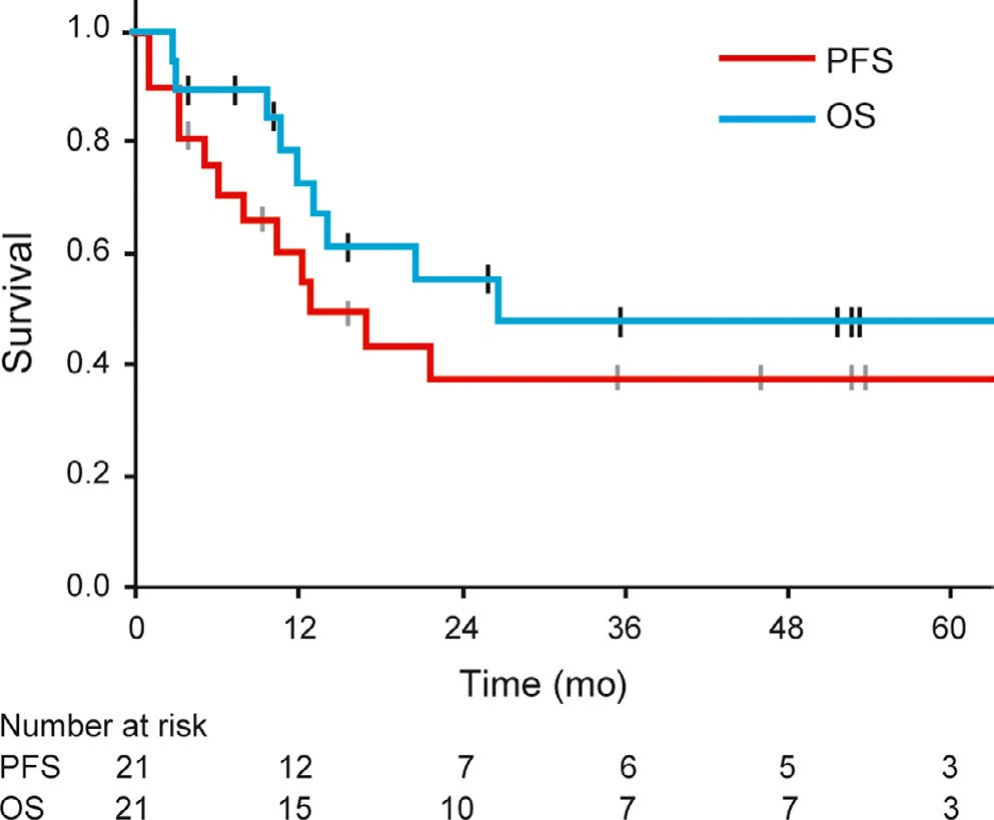
Kaplan‐Meier plots of overall survival (OS) and progression‐free survival (PFS). With a median follow‐up period of 15.5 mo, the 3‐y OS and PFS rates were 49.2% and 37.0% respectively

As shown in Table 1, 9 patients (43%) died of their disease, and one (5%) of unrelated causes within the observation period. Eleven patients (52%) developed distant metastasis, of whom 7 underwent additional treatment and 4 received best supportive care. As for the recurrence patterns in the 12 patients with recurrence, distant metastasis was noticed in 11 patients (92%), and local recurrence in only one (8%). The median duration from carbon‐ion radiotherapy initiation to the development of distant metastasis in the 11 patients was 6.0 months (range 0.9‐21.4 months). As a result, the estimated median time to recurrence for all the patients was 12.7 months (95% CI: 4.0‐21.3 months) (Figure 3). Major additional treatments for distant metastasis, especially since 2014, involve the administration of immune checkpoint inhibitors—either nivolumab or ipilimumab, or both—and these were used in 5 of our patients. In one case, nivolumab showed prolonged effects (duration longer than 2 years) on multiple liver metastasis.

The 3‐year OS rate for all the patients was 49.2% (95% CI: 25.2%‐73.2%), and the expected median OS was 26.9 months (95% CI, not estimable) (Figure 3). The 2‐year and 5‐year OS rates were 56.2% (95% CI: 33.0%‐79.3%) and 49.2% (95% CI: 25.2%‐73.2%) respectively (Figure 3). Three patients survived for more than 5 years. The 2‐year and 5‐year PFS rates were both 37.0% (95% CI: 14.6%‐59.4%) (Figure 3). The results of the univariate analysis of the prognostic factors for PFS and OS are shown in Table 3. The completion of 3 cycles of DAV therapy was not significantly associated with PFS and OS, as well as other clinical factors such as age and gender. Predictably, T4b stage and tumor invasions into deep facial tissues such as the orbit or pterygopalatine fossa were also significant prognostic factors for poor OS.

**Table 3:** Univariate analysis of survival

Characteristics	n = 21	PFS		OS	
		3‐y (%)	*P* value	3‐y (%)	*P* value
Age (y)					
≤65	11	39	.62	59	.39
>65	10	35		40	
Gender					
Male	13	44	.69	56	.55
Female	8	22		29	
T stage					
T4a	19	41	.09	55	<.01
T4b	2	0		0	
Tumor site					
Nasal cavity	16	37	.54	53	.33
Others	5	40		40	
Tumor invasion					
Orbit					
No	13	56	<.05	72	<.05
Yes	8	0		19	
Pterygopalatine fossa					
No	17	46	<.01	63	<.01
Yes	4	0		0	
Skin					
No	18	31	.26	46	.45
Yes	3	67		67	
DAV cycles					
3	16	45	.19	55	.48
≤2	5	20		30	

Abbreviation: DAV, dacarbazine, nimustine and vincristine.

## 4 DISCUSSION

The present study prospectively evaluated the efficacy and safety of the combination of carbon‐ion radiotherapy and DAV therapy in improving the prognoses of patients with mucosal melanoma of the head and neck. The high 3‐year local control rates (greater than 90%) and acceptable adverse event occurrence rates observed in this study suggest that carbon‐ion radiotherapy in combination with concurrent DAV therapy has the potential to be used in the treatment of mucosal melanoma of the head and neck even in advanced T4 stage cases, consistent with previous reports.^11^, ^18^ The 2‐ and 5‐year survival rates in this study were also comparable with those observed in the J‐CROS study—a large, multicenter, retrospective study conducted on 260 mucosal melanoma patients who underwent carbon‐ion radiotherapy in Japan.^18^ The J‐CROS study, in which 129 patients were concurrently treated by chemotherapy including DTIC, showed 2‐year and 5‐year OS rates of 69.4% and 44.6%, respectively, and univariate and multivariate analyses of their results revealed that concurrent DAV therapy was an independent prognostic factor for good OS.^18^ The corresponding OS and PFS values observed in this study, to an extent, support the efficacy of DAV therapy combined with carbon‐ion irradiation in patient survival. Clinical outcomes for mucosal melanomas using this approach compared with treatment modalities including carbon‐ions, proton beams, and photons representing conventional radiation therapy of X‐rays or cobalt‐60 in previous reports are shown in Table 4. Local control with both carbon‐ion and proton beam therapy was found to be superior to that of conventional radiotherapy, but the difference in long‐tern survival was not clear among the different modalities. In fact, the 5‐year PFS and OS rates of 37.0% and 49.2%, respectively, as observed in this study, were not satisfactory. The main reason for this discrepancy between the excellent local control and poor survival rates is the persisting high probability of distant metastasis development early after treatment. In fact, in more than 90% of the cases, distant metastasis was the observed recurrence pattern; in 82% of these cases, distant metastases developed within only 1 year after carbon‐ion radiotherapy initiation.

**Table 4:** Comparing outcomes for mucosal melanoma with treatment modalities

Author (reference)/year	Modality	n	Median follow‐up month (range)	Local control (%)	Overall survival (%)(y)
Gilligan et al^32^/1991	Photon	28	N/A	61	17 (5‐y)
Wada et al^4^/2004	Photon	31	N/A	58	33^a^, ^a^ (3‐y)
Temam et al^6^/2005	Surgery ± Photon	69	45 (8‐384)	46	47 (2‐y), 20 (5‐y)
Demizu et al^33^/2014	Proton beam	33	18.0 (6.3‐28.9)	83	91 (1‐y), 58 (2‐y)
Fuji et al^34^/2014	Proton beam	20	35 (6‐77)	80	68 (3‐y), 54 (5‐y)
Zenda et al^35^/2016	Proton beam	32	36.2	75.8	46.1 (3‐y)
Mohr et al^36^/2015	Carbon‐ion	18	18 (5‐48)	77.7	32.3 (2‐y), 16.2 (3‐y)
Koto et al^18^/2017	Carbon‐ion	260	22 (1‐132)	83.9	69.4 (2‐y), 44.6 (5‐y)

Abbreviation: N/A, not available.

^a^ Cause‐specific survival; Photon including X‐ray and cobalt‐60

The efficacy of DTIC for cutaneous melanoma has been demonstrated in some reports.^19^, ^20^ This study was initiated in April 2012, at which time DAV therapy was widely used for malignant melanoma in Japan, especially as a postoperative adjuvant chemotherapy regimen.^13^ Thereafter, a large retrospective study conducted on 142 patients with stage II or III cutaneous melanoma in Japan showed that DAV therapy does not improve survival in postoperative melanoma cases.^21^ Taking other controversial reports into consideration,^22^, ^23^, ^24^ the efficacy of DAV therapy for melanoma remains equivocal.

In contrast, the discovery of immune checkpoint inhibitors, such as ipilimumab (anti‐CTLA‐4 antibody), as well as nivolumab and pembrolizumab (anti‐PD‐1 antibodies), has revolutionized the treatment of advanced and recurrent melanoma in recent years.^25^ Pembrolizumab contributed to a significantly prolonged PFS and OS compared with ipilimumab in the KEYNOTE‐006 randomized phase III trial,^26^ and survivals of nivolumab alone or in combination with ipilimumab were superior to that of ipilimumab monotherapy in the CheckMate‐067 randomized phase III trial.^27^ Based on the results of those clinical trials, at present, nivolumab monotherapy, pembrolizumab monotherapy and nivolumab combined with ipilimumab are the first choice of systemic immunotherapy in patients with advanced melanoma.

The above‐mentioned evidence suggests the possibility that adjuvant immunotherapy with nivolumab or pembrolizumab may be more effective than concurrent and adjuvant chemotherapy with DAV in the case of carbon‐ion radiotherapy for mucosal melanoma. In cutaneous melanoma cases, adjuvant immunotherapy yields significantly greater survival in patients with postoperative stage III melanoma.^28^, ^29^, ^30^ The 3‐year PFS rate of 40%, as observed in this study, suggests that 60% of the patients showed failure following carbon‐ion radiotherapy; 30% of these cases may have been rescued by immunotherapy, as the administration of nivolumab or pembrolizumab for advanced melanoma has been shown to result in 2‐ and 3‐year PFS rates of 30%~ in clinical trials.^26^, ^27^ In fact, in this study, immunotherapy was provided as additional treatment for distant metastasis in 5 patients, of whom one with multiple liver metastasis showed almost complete response and was alive without any other recurrence for more than 2 years. Therefore, carbon‐ion radiotherapy combined with immunotherapy instead of concurrent and adjuvant DAV therapy may be the future clinical choice for advanced mucosal melanoma, as it can allow for a survival rate of 50%.

Although the maximum dose constraint to the optic nerve was set at 57 Gy (RBE), this constraint was not achieved in some patients due to advanced disease (T4a: 19 patients, 90.5%; T4b: 2 patients, 9.5%). We kept the D max to the contralateral optic nerve less than the constraint (range 0‐46Gy [RBE]). Also, we informed these patients about visual loss before carbon‐ion radiotherapy.

This study has several limitations. The sample size was a total of 21 patients which might be considered a small study population. The population was small since mucosal melanomas of the head and neck are very rare. In addition, unresectable yet clinically localized tumors requiring carbon‐ion radiotherapy are even more rare. A good general condition was also required for chemotherapy, which narrowed our study cohort even further. Furthermore, alternative treatment choice availability, such as immunotherapy for unresectable melanoma, likely reduced newly enrolled patients to this trial. In fact, the number of enrolled patients was 15 for the 3 years between 2012 and 2014, whereas it was only 6 for the 4 years between 2015 and 2019 after the first approval of nivolumab. Next, the median follow‐up period of 15.5 months may have been too short for the evaluation of survival rates. In this study, the minimum follow‐up period for 2 patients was only 3 months, however, both of them died of their disease within 3 months after carbon‐ion radiotherapy. Among the total 21 patients, the follow‐up period was less than 3 years for 15 patients, during which time 9 patients (60%) died of their disease. That is, only 6 of 21 patients (29%) are still alive less than 3 years following treatment. Since there was such a poor patient prognosis, it was difficult to attain median follow‐up period greater than 3 years for all patients. Therefore, based on the median follow‐up period of 31.2 months for all surviving patients, the 3‐year survival rates were considered evaluable in this study. However, the follow‐up durations for the surviving patients may still be insufficient for the assessment of late adverse events after carbon‐ion radiotherapy. Late toxicities such as osteonecrosis or brain necrosis could develop over several years after irradiation.^31^ Thus, further observation is desired.

In conclusion, carbon‐ion radiotherapy combined with DAV therapy led to the achievement of excellent local control for advanced mucosal melanoma of the head and neck with acceptable toxicities; however, the efficacy of additional DAV therapy in terms of survival was not sufficient, owing to distant metastases, which continued to occur frequently. Further studies on carbon‐ion radiotherapy in combination with some concomitant systemic therapies including immunotherapy should be explored to improve survival in such settings.

## CONFLICT OF INTEREST

The authors have no conflict of interest.
